# Nutrition and Physical Therapy: A Position Paper by the Physical Therapist Section of the Japanese Association of Rehabilitation Nutrition (Secondary Publication)

**DOI:** 10.31662/jmaj.2021-0201

**Published:** 2022-03-04

**Authors:** Tatsuro Inoue, Yuki Iida, Kohei Takahashi, Kengo Shirado, Fumihiko Nagano, Shinjiro Miyazaki, Izumi Takeuchi, Yoshihiro Yoshimura, Ryo Momosaki, Keisuke Maeda, Hidetaka Wakabayashi

**Affiliations:** 1 Department of Physical Therapy, Niigata University of Health and Welfare, Niigata, Japan; 2 Department of Physical Therapy, Toyohashi SOZO University School of Health Sciences, Aichi, Japan; 3 Department of Rehabilitation, Tamura Surgical Hospital, Kanagawa, Japan; 4 Department of Rehabilitation, Iizuka Hospital, Fukuoka, Japan; 5 Department of Rehabilitation, Kumamoto Rehabilitation Hospital, Kumamoto, Japan; 6 Rehabilitation Center, KKR Takamatsu Hospital, Kagawa, Japan; 7 Department of Rehabilitation, Suizenji Tohya Hospital, Kumamoto, Japan; 8 Center for Sarcopenia and Malnutrition Research, Kumamoto Rehabilitation Hospital, Kumamoto, Japan; 9 Department of Rehabilitation Medicine, Mie University Graduate School of Medicine, Mie, Japan; 10 Department of Geriatric Medicine, Hospital, National Center for Geriatrics and Gerontology, Aichi, Japan; 11 Department of Rehabilitation Medicine, Tokyo Women’s Medical University Graduate School of Medicine, Tokyo, Japan

**Keywords:** Undernutrition, Sarcopenia, Frailty, Rehabilitation nutrition, Nutritional physical therapy

## Abstract

Several patients undergoing physical therapy have nutritional problems. Knowledge of nutrition is necessary for addressing nutritional problems, such as malnutrition, sarcopenia, frailty, and cachexia. However, the relationship between physical therapy and nutrition is not fully understood. Physical therapy plays an important role in nutritional management, and evaluations, such as muscle strength and muscle mass evaluations, play an important role in nutritional screening and diagnosis. Exercise, as the core of physical therapy, is essential for nutritional interventions. Several recent studies have suggested that a combination of nutrition and physical therapy interventions can maximize the function, activity, participation, and quality of life of patients. The combination of nutrition and physical therapy interventions is key to addressing the needs of modern and diverse populations. This position paper was developed by the Physical Therapist Section of the Japanese Association of Rehabilitation Nutrition in consultation with the Japanese Society of Nutrition and Swallowing Physical Therapy.

## 1. Introduction

In recent years, there has been an increase in focus on the role of physical therapy in nutritional management because several patients undergoing physical therapy have nutritional problems. In Japan, the aging population is rapidly increasing, and several older patients undergoing physical therapy have nutritional problems, such as undernutrition, sarcopenia, and frailty. Knowledge of nutrition is, therefore, necessary for physical therapy for obesity in midlife, cachexia caused by chronic wasting disease, and athletes in the sports field. Several studies have suggested that these nutritional problems negatively affect the effectiveness of physical therapy. Thus, nutritional management is essential to maximizing the effectiveness of physical therapy.

Physical therapy plays an important role in nutritional management. However, its role in nutrition remains unclear. Clarifying the relationship between physical therapy and nutrition helps all medical professionals involved in physical therapy share common understanding and maximize function, activity, participation, and quality of life (QOL). We hope that this paper provides common understanding among all medical professionals in physical therapy and a basis for administering physical therapy to improve the outcomes of patients with nutritional problems.

## 2. The Relationship between Physical Therapy and Nutrition.

### 2-1 Physical therapy and nutrition in the world

The American Physical Therapy Association (APTA) has identified the involvement of physical therapists in nutrition and food intake as important for public health and beneficial to patients ^(1)^. However, there is no dedicated physical therapy system for nutrition, there is insufficient education and training on providing advice on the best foods for the nutritional and health statuses of a patient, and there is no official nutrition section for the physical therapy community in Japan. A certified nutritional physical therapy (CNPT^Ⓡ^) system has been established to improve the knowledge and skills of nutrition advisors in the United States ^(2)^. To be certified as a CNPT^Ⓡ^, physical therapists must complete all three of the APTA-approved education courses and must pass the certification exam. Physical therapists need to develop skills in nutritional screening and provide information to patients for appropriate feeding ^(3)^, and the scope of teaching regarding feeding and nutrition that should be carried out by physical therapists needs to be clarified and standardized ^(4)^. Thus, it has been emphasized that physical therapists should know regarding nutrition and feeding in the United States.

However, this is inconsistent with the perspectives of “physical therapy in consideration of nutrition” and “nutrition in consideration of physical therapy” found in “Rehabilitation Nutrition” in Japan. Nutritional knowledge has been highlighted among the recommended skills that a physical therapist should have. However, there are no indications on how to tailor physical therapy interventions based on nutritional status. The synergistic effects of physical therapy and nutrition on sarcopenia have been demonstrated in Europe and other countries, and there have been several reports of improved physical function, especially in patients with advanced cancer ^(5)^. However, all of these reports were based on small samples, and there have been no systematic reviews or clinical guidelines. To date, a system combining nutrition and physical therapy has not been established. In the future, it is expected that the concept of nutritional physical therapy based on rehabilitation nutrition will be globalized.

### 2-2 Physical therapy and nutrition in Japan

In Japan, it has become important for physical therapists to be involved in nutritional management. The Japan Physical Therapy Association (JPTA) has been developing an educational system for nutrition (Table 1). The JPTA established a section on nutrition and swallowing physical therapy in 2015, which was developed by the Japan Society of Nutrition and Swallowing Physical Therapy in April 2021 to further promote research and clinical practice involving nutrition and physical therapy. Nutrition has become mandatory in the pregraduate educational curriculum for physical therapist students entering in 2021 ^(6)^. For the national examination for physical therapists, questions that require knowledge of nutrition and geriatric nutritional problems such as sarcopenia and frailty are increasing. Physical therapists can obtain certifications, such as the Japanese Association of Rehabilitation Nutrition (JARN) certified rehabilitation nutritionist ^(7)^ and the Japan Society of Clinical Nutrition and Metabolism (JSPEN) certified Nutrition Support Team (NST) therapist. Physical therapists can also obtain certifications as rehabilitation nutrition instructors certified by the JARN and as NST specialists certified by the Japanese Society of Clinical Nutrition and Metabolism ^(8)^. Physical therapists are participating in the NST and are increasingly involved in nutritional management. Interventions based on both nutrition and physical therapy play an important role in meeting the varying needs of patients.

**Table 1.** Trends in Organizations and Medical Fee Revisions Related to Physical Therapy and Nutrition.

Pregraduate education curriculum for physical therapist and occupational therapist	・Required courses in nutrition (from students enrolled in 2021)
Revision of medical fees	・To enhance nutritional management in the rehabilitation wards, the participation of dietitians in rehabilitation plans is mandatory (from 2018)
	・Assignment of a full-time, dedicated dietitian in the rehabilitation wards (from 2020)
The Japan Physical Therapy Association	・Established the Section of Nutrition and Swallowing Physical Therapy (2015)
	・Developed into the Japan Society of Nutrition and Swallowing Physical Therapy (2021)
The Japan Society of Clinical Nutrition and Metabolism	・Physical therapists, occupational therapists, and speech-language therapists can obtain Nutrition Support Team (NST) Therapist (from 2010)
The Japanese Association of Rehabilitation Nutrition	・TNT-rehabilitation (from 2019), Rehabilitation nutrition instructor (from 2019)

Rehabilitation nutrition ^(9)^ has facilitated the coexistence of nutrition and physical therapy in Japan. Rehabilitation nutrition is closely related to physical therapy, in that it is based on the International Classification of Functioning, Disability, and Health (ICF). Goal setting based on the consideration of nutritional disorders, sarcopenia, and excessive or inadequate nutritional intake has been accepted in administering physical therapy regardless of the disease. With the widespread adoption of rehabilitation nutrition, the effectiveness of interventions that combine rehabilitation and nutrition has been reported ^(10), (11)^. The importance of nutritional management in rehabilitation has also been reflected in the 2018 revision of medical fees, and the participation of dietitians in the rehabilitation plan is mandatory in rehabilitation wards in Japan ^(12)^. The JARN has published medical guidelines in an international journal ^(13)^ and initiated total nutrition therapy rehabilitation to contribute to the development of academic culture and the improvement of medicine and healthcare. Thus, to clarify the effect of physical therapy on nutrition, it is necessary to understand the concept of rehabilitation nutrition.

## 3. The Effect of Physical Therapy on Nutrition

### 3-1 Concept of rehabilitation nutrition

Rehabilitation nutrition is “nutrition from the perspective of rehabilitation” or “rehabilitation from the perspective of nutrition” to improve nutritional status, sarcopenia, nutrient intake, and frailty of disabled and frail older patients and to maximize their function, activity, participation, and QOL after holistic evaluation based on ICF, assessment of the presence and causes of nutritional disorders, sarcopenia, and excess or deficient nutrient intake, diagnosis, and goal setting ^(14)^.

The effect of physical therapy on nutrition can be considered based on rehabilitation nutrition. Physical therapy can be involved in all aspects of rehabilitation nutrition. In addition to rehabilitation nutrition assessment and diagnoses, physical therapy evaluation plays a major role in rehabilitation nutrition monitoring. Physical therapists need to be involved in setting specific and achievable goals in collaboration with other medical professionals. Exercise, as the core of physical therapy, is an indispensable component of rehabilitation nutrition intervention. It is necessary for all medical professionals involved in physical therapy to share common understanding of its role in the rehabilitation nutrition care process.

### 3-2 Concept of nutritional physical therapy

**Figure 1.**
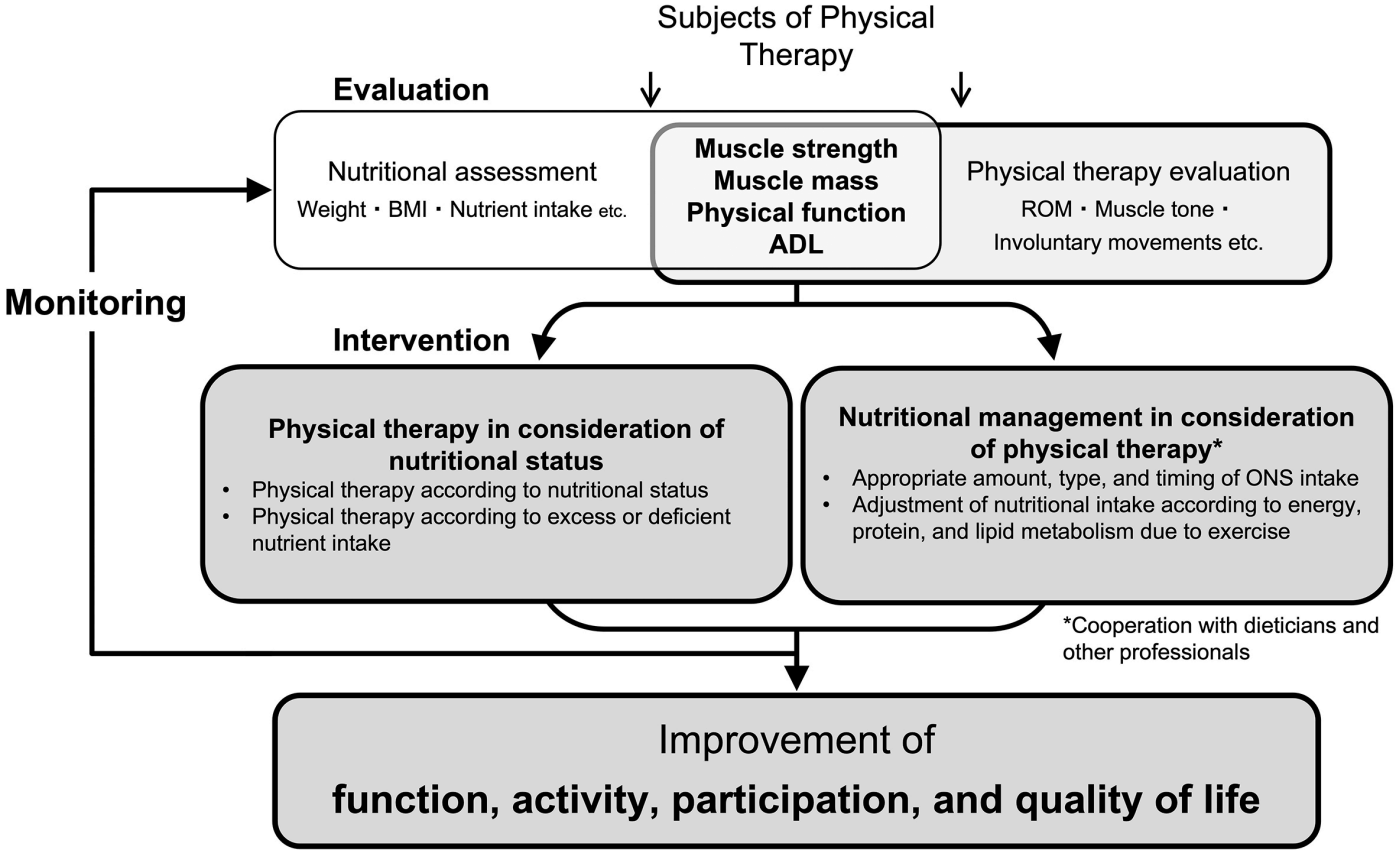
Conceptual diagram for nutritional physical therapy.

**Figure 2.**
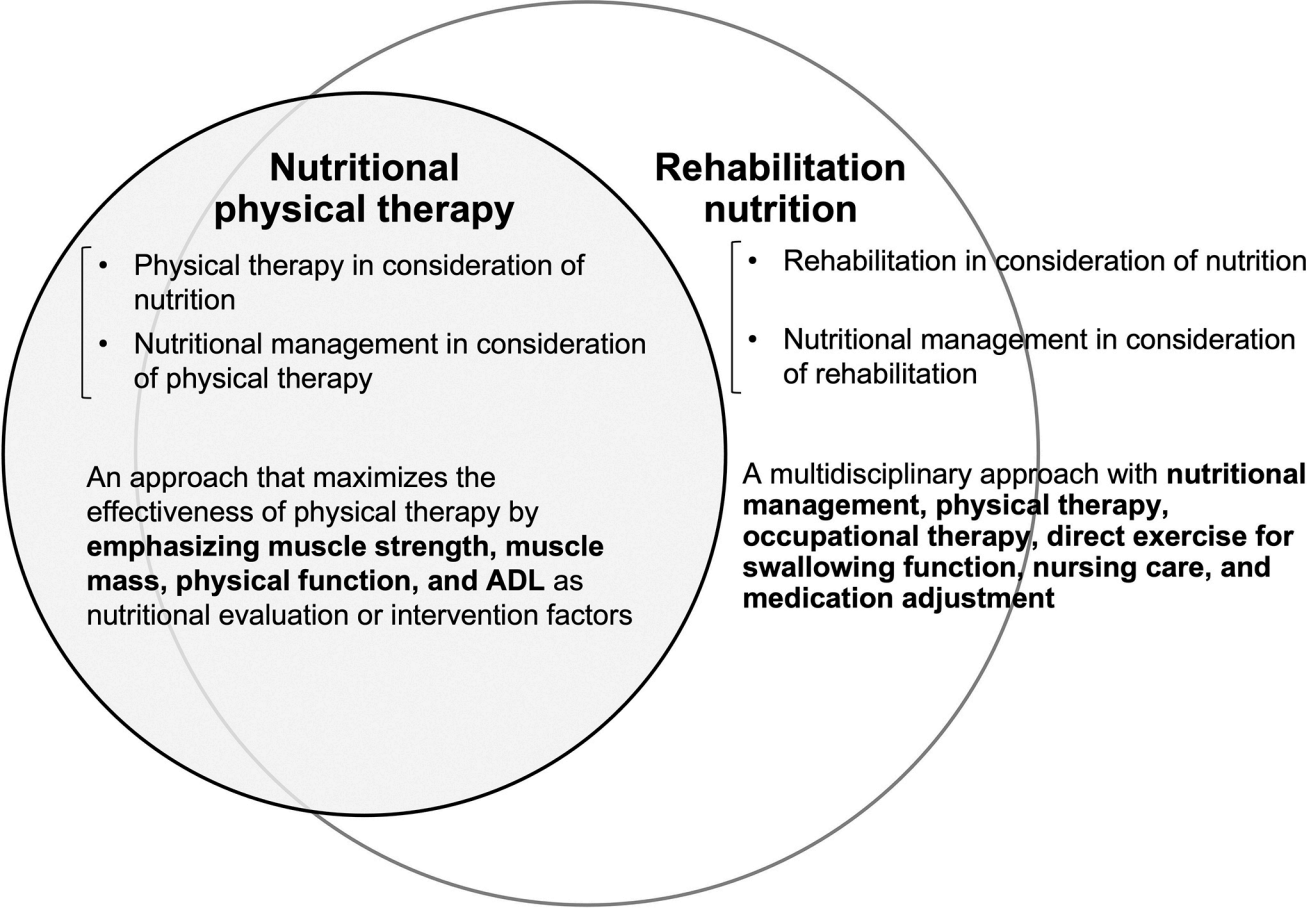
Relationship between “nutritional physical therapy” and “rehabilitation nutrition” “nutritional physical therapy” and “rehabilitation nutrition” are overlapping. The two concepts share goals, most subjects, evaluations, and intervention strategies. On the other hand, occupational therapy, direct exercise for swallowing function, nursing care, and medication adjustment are not included in “nutritional physical therapy.” The approach to support an athlete’s health is present in “nutritional physical therapy” only.

Nutritional physical therapy is physical therapy that aims to maximize the body functions, activities, participation, and QOL of people by identifying nutritional disorders, sarcopenia, and excessive or deficient nutrient intake and by setting suitable goals. For this purpose, physical therapists, dietitians, and other medical professionals conduct common nutritional and physical therapy evaluations, as well as practice nutritional management that considers physical conditions (e.g., physical activity, muscle tone, and involuntary movements) ^(15)^. In the practice of nutritional physical therapy, it is important to have the viewpoint of both “physical therapy in consideration of nutritional status” and “nutrition care management that can maximize the effect of physical therapy” (Figure 1). Nutritional physical therapy and rehabilitation nutrition are overlapping (Figure 2). The two concepts share goals, most subjects, evaluations, and intervention strategies. Nutritional physical therapy is a concept that emphasizes on muscle strength, muscle mass, physical function, and ADL for nutritional evaluation and intervention. Nutritional physical therapy and rehabilitation nutrition should be integrated to maximize the function, activity, participation, and QOL of the patient. On the other hand, occupational therapy, direct exercise for swallowing function, nursing care, and medication adjustment are not included in nutritional physical therapy.

Physical therapists need to assess the nutritional status of patients. Nutritional disorders, sarcopenia, and inadequate energy intake adversely affect the recovery of physical function ^(16), (17), (18)^. Regardless of patients receiving physical therapy often have these nutritional problems, they can be overlooked. Nutrition screening by physical therapists can identify patients with nutritional disorders ^(19), (20)^. The validated nutritional screening tools are the Mini Nutritional Assessment Short-Form (MNA^Ⓡ^-SF), Malnutrition Universal Screening Tool, and Malnutrition Screening Tool. Physical therapists can use these nutritional screening tools to screen for nutritional status. If malnutrition is suspected, physical therapists should consult a nutrition professional (e.g., nutritionist and specialist clinician) to further evaluate nutritional status and consider appropriate nutritional care management. Multimodal nutritional interventions may reduce morbidity and mortality associated with nutritional disorders and may improve the outcomes of patients ^(20)^.

Nutritional status is associated with muscle mass and strength, physical function, and ADL. These functional measurements are increasingly important for nutritional assessment (Table 2) ^(21), (22), (23), (24)^. The diagnostic criteria for malnutrition by the American Society for Parenteral and Enteral Nutrition and the American Dietitians Association (AND) include decreased muscle mass and function based on grip strength ^(25)^. The Global Leadership Initiative on Malnutrition (GLIM) criteria also recommend that functional assessments based on hand grip strength provide a supportive measure of muscle mass ^(26)^. Muscle strength tests include grip strength, knee extensor strength, and peak expiratory flow ^(21), (24)^. Muscle function tests include walking speed, short physical performance battery, and timed up and go test ^(24)^. Muscle strength and function respond more sensitively to nutritional status than body composition and can be used to monitor nutritional management ^(21)^. In addition, ADL assessment should be used as a nutritional monitoring and outcome measure because ADL impairment results from muscle weakness ^(23)^. These assessments have been professionally assessed by physical therapists and can be used for nutritional assessment. Thus, it is important to share physical therapy evaluations with dietitians, nurses, and clinicians, which leads to appropriate nutritional management.

**Table 2.** Physical Therapy Evaluation, Nutritional Diagnosis, and Screening.

	Physical frailty	Sarcopenia		Nutritional status			Cachexia	
	J-CHS ^(53)^	AWGS 2019 ^(54)^	EWGSOP2 ^(55)^	GLIM ^(26)^	ASPEN and AND ^(25)^	MNA ^(56)^	Evans ^(57)^	Fearon ^(58)^
Muscle mass		◯	◯	◯	◯	◯	◯	◯
Muscle strength	◯	◯	◯	◯ (supplementary)	◯		◯	
Physical function	◯	◯	◯					
ADL		◯ (case finding)				◯ (gait)		
Weight loss	◯	◯ (case finding)		◯		◯	◯	◯
BMI				◯	◯	◯	◯	

AWGS, Asian Working Group for Sarcopenia; EWGSOP, European Working Group on Sarcopenia in Older People; GLIM, Global Leadership Initiative on Malnutrition; ASPEN, American Society for Parenteral and Enteral Nutrition; AND, Academy of Nutrition and Dietetics; MNA, Mini Nutritional Assessment

Nutritional management enhances the effectiveness of physical therapy, and the effectiveness of nutritional management is enhanced by physical therapy. Physical therapy combined with nutritional management may improve function, activity, and participation ^(9)^. In addition, adequate protein intake and resistance training (RT) should be combined to increase skeletal muscle mass, which is an important objective of nutritional management ^(27)^. For overnutrition and sarcopenic obesity, it is useful to combine RT and aerobic exercise instead of energy restriction alone ^(28)^. Thus, exercise is an essential component of the nutritional management of patients requiring physical therapy, and physical therapy plays an important role in multimodal nutritional management.

To implement effective nutritional physical therapy, we need to understand that age-related loss of skeletal muscle mass and muscle weakness are not exactly parallel. Previous studies have reported that age-related muscle weakness appears earlier than the loss of skeletal muscle mass ^(29)^. In addition, muscle strength is considered a better predictor of negative health outcomes than skeletal muscle mass ^(30)^. Therefore, muscle strength, including the entire neuromuscular function, is recognized as a biomarker that determines the well-being of older adults ^(31)^. On the other hand, skeletal muscle mass directly reflects nutritional status. For example, in chronic inflammatory diseases, such as chronic obstructive pulmonary disease, loss of skeletal muscle mass may be more apparent than muscle weakness, but there is no clear evidence. The response to RT is also different between muscle strength and skeletal muscle mass. The increase in skeletal muscle mass due to RT appears later than the increase in muscle strength ^(32)^. In a study of older adults who were followed up for 3 months after hospitalization, muscle strength and physical function recovered to the same level or even higher than that at the time of hospitalization. However, skeletal muscle mass did not recover during hospitalization ^(33)^. Therefore, we believe that long-term nutritional physical therapy is necessary to improve skeletal muscle mass.

#### 3-2-1 Physical therapy in consideration of nutrition

**Figure 3.**
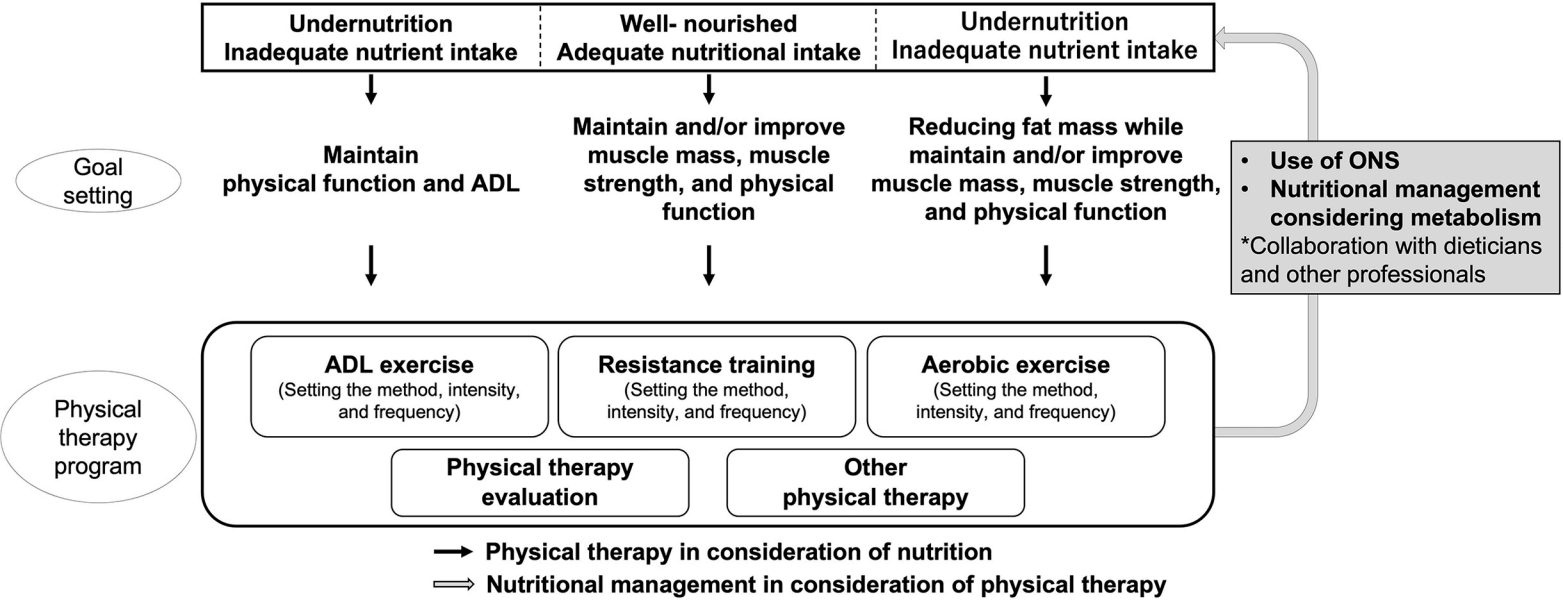
Interactive perspective on nutrition and physical therapy.

Physical therapy should be performed depending on nutritional status. Adequate nutrient intake leads to improvement and maintenance of physical function, physical activity, participation, and QOL (Figure 3). Gender-specific approaches are necessary for some diseases and conditions. For example, nutritional physical therapy with protein, calcium, and vitamin D intake combined with RT and balance exercises is recommended for older postmenopausal women at high risk for osteoporotic fractures because the decrease in estrogen production after menopause causes a decrease in bone mineral density. For effective physical therapy, nutritional status should be constantly monitored by a multidisciplinary team.

In well-nourished patients, the goal of physical therapy is to improve or maintain muscle mass, muscle strength, and physical function according to the health status and objectives of the patient. As skeletal muscle mass decreases with aging ^(34)^, older patients should prevent frailty and sarcopenia, even if they are well nourished. The effect of branched-chain amino acid intake in combination with RT improves muscle protein synthesis and muscle strength in athletes ^(35)^. Similarly, branched-chain amino acid intake combined with exercise can improve the skeletal muscle mass, muscle strength, and physical function of older patients ^(36)^.

For cases of undernutrition, physical therapy should be administered depending on the cause. If undernutrition is caused by severe invasion, the goal of physical therapy is to maintain ADL and QOL as much as possible because excessive loads lead to muscle breakdown. Highly invasive cases and nutritional disorders are more severe because of hyperglycemia and accelerated muscle breakdown ^(37)^. However, the energy expenditure associated with leaving the bed is negligible ^(38)^, and leaving the bed should be actively practiced to prevent disuse syndrome. As metabolism shifts to anabolism, skeletal muscle mass is expected to increase, and active nutritional management and physical therapy should be performed to improve nutritional status and physical function. In the case of undernutrition due to lack of nutrient intake, high-intensity exercise during starvation may accelerate muscle breakdown, but rest and calorie-restricted conditions lead to further reductions in muscle mass. Hence, medical staff should pay attention to avoid unnecessary bed rest. The exercise load should be gradually increased in parallel with improvements in nutritional status through nutritional management.

Resting may lead to obesity and overnutrition; physical therapy is mainly aerobic taking into account carbohydrate and lipid metabolism ^(39)^. For sarcopenic obesity, which increases the risk of developing cardiovascular and metabolic chronic diseases, a comprehensive approach, including nutritional therapy (moderate low energy diet and high protein intake of at least 1.2 g/kg/day) and RT, is recommended ^(28)^．

#### 3-2-2 Nutritional management in consideration of physical therapy

“Nutritional management in consideration of physical therapy” means the adjustment of the nutrient intake of energy, protein, and lipid due to exercise in consultation with multidisciplinary medical professionals. Considerations of the timing, quality, and quantity of nutrient intake are necessary when implementing RT. The promotion of muscle protein synthesis after RT should be considered, and optimal intake of protein and amino acids will improve the muscle strength and muscle mass of the patient. It has been reported that muscle protein synthesis is more enhanced after RT and nutrient intake than after nutrition intervention alone or RT alone ^(40)^, and this response is not exclusive to older adults ^(41)^. Regarding nutritional quality, it has been reported that β-hydroxy-β-methylbutyrate (HMB), in addition to leucine, which is the most potent branched-chain amino acid that stimulates muscle protein synthesis ^(42)^, is effective in increasing muscle mass in older adults ^(43)^, but the results are not consistent ^(44)^. The European Society for Clinical Nutrition and Metabolism (ESPEN) guidelines recommend a protein intake of at least 1 g/kg/day for older adults ^(45)^. The PROT-AGE Study Group position paper recommends a protein intake of 1.2-1.5 g/kg/day for older adults with acute and chronic diseases and 2.0 g/kg/day for critically ill, traumatized, or malnourished patients with consideration of renal function ^(46)^. For patients receiving RT, adding nutritional intake with appropriate timing, quality, and quantity can maximize the effect of RT. The combined use of amino acid-based nutrition and exercise has been reported to be effective in a study of older patients with sarcopenia during hospitalization ^(10), (47)^. This suggests that combined physical therapy and nutritional interventions are effective for hospitalized older adults with sarcopenia. Oral nutrition supplements are recommended depending on the patient’s general condition and swallowing ability. Previous studies have shown that leucine-rich jelly-type supplements ^(10)^ or powdered supplements dissolved in water ^(47)^ effectively increase skeletal muscle mass and muscle strength and improve ADL in older patients with sarcopenia undergoing rehabilitation.

Accurate assessment of energy metabolism resulting from exercise can lead to appropriate nutritional management. Nutritional status deteriorates if nutritional management does not consider the energy metabolism resulting from exercise. Exercise-induced energy metabolism was calculated using metabolic equivalents (METs) ^(48)^. The energy expenditure for activities for a certain period is calculated by considering the energy expenditure during the activity, in addition to the energy expenditure at rest. The total energy expenditure of hospitalized patients undergoing physical therapy should be evaluated with considerations of the amount of energy expenditure during physical therapy. The rationale for setting activity factors according to the intensity of physical therapy is unclear, but a guideline is 1.3 as an activity factor for 20 minutes of physical therapy, 1.3-1.7 for ≥1 hour, and 1.5-2.0 for ≥2 hours ^(49)^. In addition, accurate assessments of abnormal muscle tone and involuntary movements caused by cerebrovascular and neurodegenerative diseases will lead to appropriate nutritional management. It has been reported that oxygen consumption during exercise is higher in patients with Parkinson’s disease than in healthy people ^(50)^, and deep stimulation of the subthalamic nucleus increases body weight and BMI ^(51)^. In addition, it has been reported that energy expenditure is higher in patients with cerebellar ataxia than in healthy people because of decreased coordination during walking ^(52)^. Appropriate physical therapy evaluation based on disease characteristics is necessary to prevent the deterioration of nutritional status due to increased energy expenditure.

## 4. Future Perspectives

This position paper, which is the first to assess the synergistic effects of nutrition and physical therapy, provides insights into the effects of physical therapy on nutrition and vice versa based on domestic and international trends. Regardless of the disease or stage of the disease, it is necessary to focus on nutrition and physical therapy to appropriately address nutritional problems and to maintain and improve function, activity, participation, and QOL, which are the original objectives of physical therapy. On the other hand, systematic clinical practice based on an interactive perspective of nutrition and physical therapy has not been adequately implemented. In addition, it needs to simplify nutritional evaluation to perform rapid and effective intervention in any environment. Further clinical practice and research are required.

This is the secondary English version of the original Japanese manuscript for “Nutrition and physical therapy: A position paper by the physical therapist section of the Japanese Association of Rehabilitation Nutrition ^(59)^.”

## Article Information

### Conflicts of Interest

None

### Acknowledgement

We would like to thank the Japanese Society of Nutrition and Swallowing Physical Therapy for their advice in developing this position paper. We are also grateful for public comments on this paper.

### Author Contributions

Substantial contributions to the conception or design of the work: Tatsuro Inoue, Yuki Iida, Kohei Takahashi, Kengo Shirado, Fumihiko Nagano, Shinjiro Miyazaki, Izumi Takeuchi, Yoshihiro Yoshimura, Ryo Momosaki, Keisuke Maeda, and Hidetaka Wakabayashi

Drafting the work: Tatsuro Inoue, Yuki Iida, Kohei Takahashi, Kengo Shirado, Fumihiko Nagano, Shinjiro Miyazaki, Izumi Takeuchi, Yoshihiro Yoshimura, Ryo Momosaki, Keisuke Maeda, and Hidetaka Wakabayashi

Final approval of the version to be published: Tatsuro Inoue, Yuki Iida, Kohei Takahashi, Kengo Shirado, Fumihiko Nagano, Shinjiro Miyazaki, Izumi Takeuchi, Yoshihiro Yoshimura, Ryo Momosaki, Keisuke Maeda, and Hidetaka Wakabayashi

Agreement to be accountable for all aspects of the work in ensuring that questions related to the accuracy or integrity of any part of the work are appropriately investigated and resolved: Tatsuro Inoue, Yuki Iida, Kohei Takahashi, Kengo Shirado, Fumihiko Nagano, Shinjiro Miyazaki, Izumi Takeuchi, Yoshihiro Yoshimura, Ryo Momosaki, Keisuke Maeda, and Hidetaka Wakabayashi

### Approval by Institutional Review Board (IRB)

Not applicable.

* The editor-in-chiefs of the Journal of the Japanese Association of Rehabilitation Nutrition and JMA Journal have given permission for secondary publication of this position paper.

* This article is based on a study first reported in the Journal of the Japanese Association of Rehabilitation Nutrition. 2021;5(2):226-234 (Japanese) ^(59)^

Journal articles published ahead of issue (print): Inoue T et al. Nutrition and physical therapy: a position paper by the physical therapist section of the Japanese Association of Rehabilitation Nutrition. The Journal of the Japanese Association of Rehabilitation Nutrition. 2021.

* The original version is available at https://sites.google.com/site/jsrhnt/ポジションペーパー?authuser=0.
